# Bis(μ-di-2-pyridyl disulfide-κ^3^ *N*,*S*:*N*′)di-μ_3_-iodido-di-μ_2_-iodido-tetra­copper(I)

**DOI:** 10.1107/S160053681105152X

**Published:** 2011-12-07

**Authors:** Yu-Hong Wang, Xue-Hua Zhu

**Affiliations:** a School of Chemistry and Bioengineering, Suzhou University of Science and Technology, Suzhou 215009, People’s Republic of China

## Abstract

In the centrosymmetric tetra­nuclear title compound, [Cu_4_I_4_(C_10_H_8_N_2_S_2_)_2_], there are two different Cu^I^ atoms with tetra­hedral coordination geometries. One is chelated by a pyridine N atom and an S-donor from one di-2-pyridyl disulfide ligand and coordinated by two I atoms, while the second Cu^I^ atom is coordinated by a pyridine-N and three I atoms. Iodine bridges between the Cu^I^ atoms form a tetra­nuclear structure.

## Related literature

For the structures and luminescence properties of Cu(I) complexes, see: Caradoc-Davies & Hanton (2003 ▶); Ford *et al.* (1999 ▶); Rath *et al.* (1986 ▶); Song *et al.* (2003 ▶); Song, Sun & Yang (2011) ▶; Song, Sun, Yang & Yang (2011 ▶); Su *et al.* (1997 ▶). For metal complexes with di-2-pyridyl disulfide, see: Bell *et al.* (2000 ▶); Delgado *et al.* (2007 ▶); Kadooka *et al.* (1976 ▶); Niu *et al.* (2007 ▶); Wu *et al.* (2011 ▶).
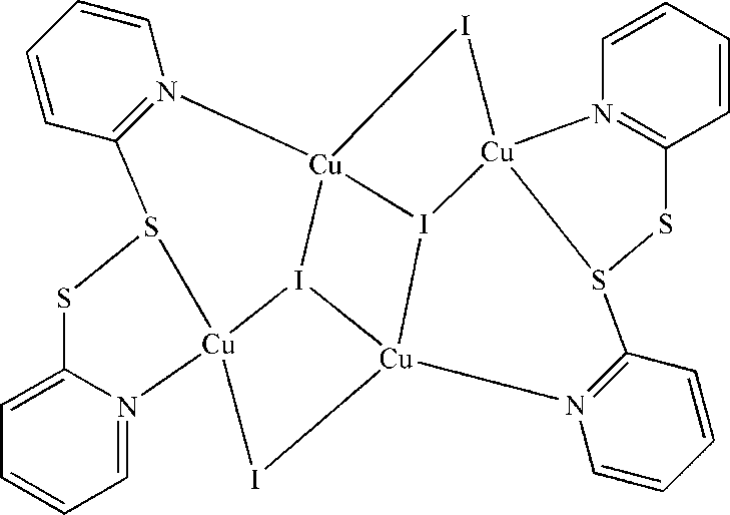


## Experimental

### 

#### Crystal data

[Cu_4_I_4_(C_10_H_8_N_2_S_2_)_2_]
*M* *_r_* = 1202.37
Orthorhombic, 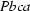

*a* = 10.460 (6) Å
*b* = 14.434 (8) Å
*c* = 19.908 (12) Å
*V* = 3006 (3) Å^3^
*Z* = 4
Mo *K*α radiation
μ = 7.20 mm^−1^
*T* = 223 K
0.38 × 0.11 × 0.10 mm

#### Data collection

Rigaku Saturn diffractometer
Absorption correction: multi-scan (*REQAB*; Jacobson, 1998 ▶) *T*_min_ = 0.171, *T*_max_ = 0.533
10455 measured reflections
3410 independent reflections
2944 reflections with *I* > 2σ(*I*)
*R*_int_ = 0.041

#### Refinement

*R*[*F* ^2^ > 2σ(*F* ^2^)] = 0.048
*wR*(*F* ^2^) = 0.087
*S* = 1.16
3410 reflections
163 parameters
H-atom parameters constrained
Δρ_max_ = 0.73 e Å^−3^
Δρ_min_ = −0.68 e Å^−3^

### 

Data collection: *CrystalClear* (Rigaku, 2001 ▶); cell refinement: *CrystalClear*; data reduction: *CrystalStructure* (Rigaku, 2001 ▶); program(s) used to solve structure: *SHELXS97* (Sheldrick, 2008 ▶); program(s) used to refine structure: *SHELXL97* (Sheldrick, 2008 ▶); molecular graphics: *SHELXTL* (Sheldrick, 2008 ▶); software used to prepare material for publication: *SHELXTL*.

## Supplementary Material

Crystal structure: contains datablock(s) I, global. DOI: 10.1107/S160053681105152X/mw2029sup1.cif

Structure factors: contains datablock(s) I. DOI: 10.1107/S160053681105152X/mw2029Isup2.hkl

Additional supplementary materials:  crystallographic information; 3D view; checkCIF report

## Figures and Tables

**Fig. 1.**
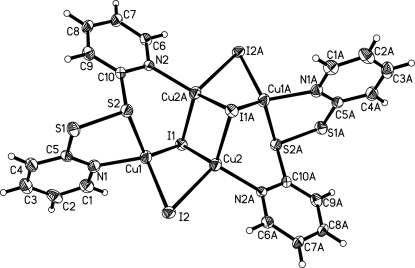
View of (I) shown with 30% probability displacement ellipsoids and small spheres for the H atoms.

## supplementary crystallographic information

### Comment

Polynuclear Cu^I^ compounds with luminescent properties have been designed and synthesized by reaction of Cu^I^ cations and suitable bridging ligands (Ford *et al.*, 1999). Flexible heterocyclic ligands containing nitrogen donors can form metal complexes with considerable structural diversity and reports of Cu^I^ complexes with such ligands have increased in recent years (Caradoc-Davies & Hanton, 2003; Rath *et al.*, 1986; Song *et al.*, 2003; Song, Sun & Yang, 2011; Song, Sun, Yang & Yang, 2011; Song, Sun, & Yang, 2011; Su *et al.*, 1997). di-2-pyridyl disulfide is a interesting heterocyclic ligand. A number of metal complexes of it and related ligands have been reported (Bell *et al.*, 2000; Delgado *et al.*, 2007; Kadooka *et al.*, 1976; Niu *et al.*, 2007; Wu *et al.*, 2011). The present study details the structure of a CuI adduct of di-2-pyridyl disulfide.

The title compound, (I), is a tetranuclear complex, [Cu_4_I_4_(*L*)_2_] having crystallographically-imposed centrosymmetry (Fig. 1). In the unique ligand, S(2) and N(1) chelate to Cu(1) while N(2) coordinates to Cu(2A). Doubly-bridging I2 and triply-bridging I1 serve to complete the formation of the tetranuclear core in which each copper adopts approximately tetrahedral geometry. Cu(1) is coordinated by a pyridine N atom and an S donor from one *L* ligand and two iodine atoms while Cu(2) is coordinated by a pyridine N atom from the second *L* ligand and three iodine atoms. The bond angles around Cu(2) range from 104.38 (4) to 114.42 (5)° and are normal for a slightly distorted tetrahedral geometry while Cu(1) exists in a more distorted tetrahedral environment with the angles around Cu(1) in the range 88.58 (17) to 122.55 (7)°. All the Cu—N and Cu—S bond distances are comparable with those of other tetrameric clusters (Rath *et al.*, 1986; Song *et al.*, 2003; Su *et al.*, 1997). The Cu—I bond lengths show significant differences depending on the Cu(I) environment. Cu(2) bonds to three iodine atoms with Cu—I bond distances ranging from 2.6864 (15) to 2.7623 (17) Å, while Cu(1) bonds to two iodine atoms with equivalent shorter Cu—I bond distances averaging 2.5764 (14) Å. The Cu···Cu distance of 2.6648 (18) Å is shorter than the sum of their van der Waals radii (2.83 Å), indicating the existence of Cu···Cu interactions. Such short Cu···Cu separations have been observed in some complexes such as [Cu_4_I_4_(quin)_4_] [2.582 (10)Å](Rath *et al.*, 1986), [Cu_4_I_4_(MPTQ)_2_] [2.607 (1)Å] (Su *et al.*, 1997), [Cu_4_I_4_(C_19_H_36_N_2_S_2_)_2_]_n_ [2.734 (2)Å] (Song, Sun & Yang, 2011), [Cu_4_I_4_(C_15_H_12_N_2_S)_2_] [2.743 (1)Å] (Song *et al.*, 2003) and [CuI(bbbm)]_n_ [2.7683 (9)Å] (Niu *et al.*, 2007).

The tetrameric Cu_4_I_4_ core adopts a distorted chair-like structure which is defined by three Cu—I edges from different Cu_2_I_2_ moieties: the strictly planar Cu(2)—I(1A)—Cu(2A)—I(1) unit and the non-planar Cu(1)—I(2)—Cu(2)—I(1) and symmetry-related Cu(1A)—I( A)—Cu(2A)—I(1A) units. Similar results were also found in related tetranuclear complexes (Rath *et al.*, 1986; Song *et al.*, 2003; Su *et al.*, 1997).

### Experimental

The ligand di-2-pyridyl disulfide (22.0 mg, 0.1 mmol) in CHCl_3_ (3 ml) was added to CuI (17.8 mg, 0.1 mmol) dissolved in MeCN (5 ml). The reaction mixture was kept in the dark and allowed to evaporate slowly. Yellow block-shaped single crystals suitable for X-ray analysis were obtained in 58% yield. Analysis found: C 20.39, H 1,40, N 4.84%; calculated for C_20_H_16_Cu_4_I_4_N_4_S_4_: C 19.98, H 1.34, N 4.66%.

### Refinement

H atoms were placed in calculated positions and included as riding contributions with C—H distances of 0.94Å (aromatic H) and with *U*_iso_= 1.2*U*_eq_(C).

### Crystal data

**Table:**

[Cu_4_I_4_(C_10_H_8_N_2_S_2_)_2_]	*F*(000) = 2224
*M**_r_* = 1202.37	*D*_x_ = 2.657 Mg m^−^^3^
Orthorhombic, *P**b**c**a*	Mo *K*α radiation, λ = 0.71075 Å
Hall symbol: -P 2ac 2ab	Cell parameters from 8826 reflections
*a* = 10.460 (6) Å	θ = 3.0–27.5°
*b* = 14.434 (8) Å	µ = 7.20 mm^−^^1^
*c* = 19.908 (12) Å	*T* = 223 K
*V* = 3006 (3) Å^3^	Block, yellow
*Z* = 4	0.38 × 0.11 × 0.10 mm

### Data collection

**Table:**

Rigaku Saturn diffractometer	3410 independent reflections
Radiation source: fine-focus sealed tube	2944 reflections with *I* > 2σ(*I*)
graphite	*R*_int_ = 0.041
Detector resolution: 14.63 pixels mm^-1^	θ_max_ = 27.5°, θ_min_ = 3.0°
ω scans	*h* = −9→13
Absorption correction: multi-scan (*REQAB*; Jacobson, 1998)	*k* = −11→18
*T*_min_ = 0.171, *T*_max_ = 0.533	*l* = −23→25
10455 measured reflections	

### Refinement

**Table:**

Refinement on *F*^2^	Primary atom site location: structure-invariant direct methods
Least-squares matrix: full	Secondary atom site location: difference Fourier map
*R*[*F*^2^ > 2σ(*F*^2^)] = 0.048	Hydrogen site location: inferred from neighbouring sites
*wR*(*F*^2^) = 0.087	H-atom parameters constrained
*S* = 1.16	*w* = 1/[σ^2^(*F*_o_^2^) + (0.0286*P*)^2^] where *P* = (*F*_o_^2^ + 2*F*_c_^2^)/3
3410 reflections	(Δ/σ)_max_ = 0.001
163 parameters	Δρ_max_ = 0.73 e Å^−^^3^
0 restraints	Δρ_min_ = −0.68 e Å^−^^3^

### Special details

**Table:**

Geometry. All e.s.d.'s (except the e.s.d. in the dihedral angle between two l.s. planes) are estimated using the full covariance matrix. The cell e.s.d.'s are taken into account individually in the estimation of e.s.d.'s in distances, angles and torsion angles; correlations between e.s.d.'s in cell parameters are only used when they are defined by crystal symmetry. An approximate (isotropic) treatment of cell e.s.d.'s is used for estimating e.s.d.'s involving l.s. planes.
Refinement. Refinement of *F*^2^ against ALL reflections. The weighted *R*-factor *wR* and goodness of fit *S* are based on *F*^2^, conventional *R*-factors *R* are based on *F*, with *F* set to zero for negative *F*^2^. The threshold expression of *F*^2^ > σ(*F*^2^) is used only for calculating *R*-factors(gt) *etc*. and is not relevant to the choice of reflections for refinement. *R*-factors based on *F*^2^ are statistically about twice as large as those based on *F*, and *R*- factors based on ALL data will be even larger.

### Fractional atomic coordinates and isotropic or equivalent isotropic displacement parameters (Å^2^)

**Table:**

	*x*	*y*	*z*	*U*_iso_*/*U*_eq_	
I1	0.28885 (4)	0.00696 (3)	0.52344 (2)	0.03779 (13)	
I2	0.56758 (4)	0.03336 (3)	0.68154 (2)	0.04152 (14)	
Cu1	0.43879 (8)	0.11605 (5)	0.58888 (4)	0.0440 (2)	
Cu2	0.52112 (8)	−0.05428 (6)	0.56063 (5)	0.0430 (2)	
S1	0.55004 (18)	0.33204 (12)	0.60075 (10)	0.0472 (5)	
S2	0.57511 (16)	0.22524 (11)	0.53450 (9)	0.0381 (4)	
N1	0.3452 (5)	0.2256 (4)	0.6339 (3)	0.0409 (13)	
N2	0.4533 (4)	0.1941 (3)	0.4210 (3)	0.0300 (11)	
C1	0.2348 (6)	0.2116 (5)	0.6675 (4)	0.0504 (19)	
H1	0.1960	0.1530	0.6644	0.061*	
C2	0.1761 (7)	0.2779 (6)	0.7059 (4)	0.058 (2)	
H2	0.1002	0.2647	0.7293	0.069*	
C3	0.2316 (7)	0.3650 (6)	0.7093 (4)	0.057 (2)	
H3	0.1933	0.4124	0.7347	0.069*	
C4	0.3452 (7)	0.3811 (5)	0.6745 (4)	0.0514 (19)	
H4	0.3844	0.4397	0.6756	0.062*	
C5	0.3986 (6)	0.3093 (4)	0.6386 (3)	0.0400 (16)	
C6	0.3900 (6)	0.2177 (4)	0.3649 (3)	0.0374 (15)	
H6	0.3637	0.1702	0.3357	0.045*	
C7	0.3617 (6)	0.3078 (4)	0.3479 (4)	0.0432 (17)	
H7	0.3157	0.3215	0.3085	0.052*	
C8	0.4027 (6)	0.3779 (4)	0.3903 (4)	0.0424 (17)	
H8	0.3860	0.4401	0.3796	0.051*	
C9	0.4671 (6)	0.3565 (4)	0.4474 (4)	0.0399 (16)	
H9	0.4960	0.4031	0.4767	0.048*	
C10	0.4893 (5)	0.2629 (4)	0.4615 (3)	0.0307 (14)	

### Atomic displacement parameters (Å^2^)

**Table:**

	*U*^11^	*U*^22^	*U*^33^	*U*^12^	*U*^13^	*U*^23^
I1	0.0346 (2)	0.0420 (3)	0.0368 (3)	−0.00164 (19)	−0.00135 (19)	−0.0036 (2)
I2	0.0539 (3)	0.0378 (2)	0.0329 (3)	−0.0041 (2)	−0.0081 (2)	0.00119 (19)
Cu1	0.0558 (5)	0.0339 (4)	0.0423 (6)	0.0019 (4)	−0.0071 (4)	−0.0047 (4)
Cu2	0.0515 (5)	0.0329 (4)	0.0447 (6)	0.0009 (4)	−0.0026 (4)	0.0002 (4)
S1	0.0593 (11)	0.0411 (10)	0.0411 (11)	−0.0087 (9)	−0.0065 (9)	−0.0111 (8)
S2	0.0420 (9)	0.0368 (9)	0.0354 (10)	0.0018 (8)	−0.0076 (8)	−0.0033 (7)
N1	0.045 (3)	0.041 (3)	0.037 (4)	0.008 (3)	−0.008 (3)	−0.001 (3)
N2	0.034 (3)	0.027 (3)	0.029 (3)	0.001 (2)	0.003 (2)	0.001 (2)
C1	0.047 (4)	0.059 (5)	0.045 (5)	−0.002 (4)	−0.005 (3)	−0.004 (4)
C2	0.049 (4)	0.089 (6)	0.036 (5)	0.016 (4)	−0.009 (4)	−0.011 (4)
C3	0.063 (5)	0.060 (5)	0.050 (5)	0.028 (4)	−0.016 (4)	−0.015 (4)
C4	0.071 (5)	0.044 (4)	0.039 (5)	0.014 (4)	−0.011 (4)	−0.008 (3)
C5	0.054 (4)	0.037 (4)	0.028 (4)	0.006 (3)	−0.010 (3)	−0.003 (3)
C6	0.037 (3)	0.041 (4)	0.033 (4)	0.005 (3)	−0.009 (3)	−0.005 (3)
C7	0.047 (4)	0.039 (4)	0.044 (4)	0.017 (3)	−0.008 (3)	0.004 (3)
C8	0.049 (4)	0.034 (3)	0.044 (4)	0.014 (3)	−0.007 (3)	0.002 (3)
C9	0.038 (3)	0.039 (4)	0.043 (4)	−0.004 (3)	0.000 (3)	−0.007 (3)
C10	0.029 (3)	0.029 (3)	0.034 (4)	0.001 (3)	0.000 (3)	−0.005 (3)

### Geometric parameters (Å, °)

**Table:**

I1—Cu1	2.5760 (13)	N2—Cu2^i^	2.068 (5)
I1—Cu2^i^	2.6868 (15)	C1—C2	1.370 (10)
I1—Cu2	2.6892 (16)	C1—H1	0.9400
I2—Cu1	2.5772 (13)	C2—C3	1.385 (11)
I2—Cu2	2.7623 (17)	C2—H2	0.9400
Cu1—N1	2.064 (5)	C3—C4	1.395 (10)
Cu1—S2	2.385 (2)	C3—H3	0.9400
Cu1—Cu2	2.6650 (18)	C4—C5	1.377 (9)
Cu2—N2^i^	2.068 (5)	C4—H4	0.9400
Cu2—I1^i^	2.6868 (15)	C6—C7	1.377 (8)
Cu2—Cu2^i^	2.912 (2)	C6—H6	0.9400
S1—C5	1.785 (7)	C7—C8	1.386 (9)
S1—S2	2.046 (3)	C7—H7	0.9400
S2—C10	1.792 (6)	C8—C9	1.357 (9)
N1—C5	1.334 (8)	C8—H8	0.9400
N1—C1	1.350 (9)	C9—C10	1.399 (8)
N2—C10	1.334 (7)	C9—H9	0.9400
N2—C6	1.341 (7)		
			
Cu1—I1—Cu2^i^	73.10 (5)	C1—N1—Cu1	120.4 (5)
Cu1—I1—Cu2	60.77 (4)	C10—N2—C6	117.0 (5)
Cu2^i^—I1—Cu2	65.59 (5)	C10—N2—Cu2^i^	125.6 (4)
Cu1—I2—Cu2	59.76 (4)	C6—N2—Cu2^i^	117.4 (4)
N1—Cu1—S2	88.52 (17)	N1—C1—C2	123.7 (7)
N1—Cu1—I1	113.53 (16)	N1—C1—H1	118.1
S2—Cu1—I1	122.57 (7)	C2—C1—H1	118.1
N1—Cu1—I2	106.96 (16)	C1—C2—C3	118.3 (8)
S2—Cu1—I2	108.56 (6)	C1—C2—H2	120.9
I1—Cu1—I2	113.40 (5)	C3—C2—H2	120.9
N1—Cu1—Cu2	162.08 (17)	C2—C3—C4	118.9 (7)
S2—Cu1—Cu2	108.70 (7)	C2—C3—H3	120.5
I1—Cu1—Cu2	61.71 (4)	C4—C3—H3	120.5
I2—Cu1—Cu2	63.57 (4)	C5—C4—C3	118.5 (7)
N2^i^—Cu2—Cu1	154.75 (15)	C5—C4—H4	120.7
N2^i^—Cu2—I1^i^	105.23 (13)	C3—C4—H4	120.7
Cu1—Cu2—I1^i^	97.82 (4)	N1—C5—C4	123.2 (7)
N2^i^—Cu2—I1	119.11 (13)	N1—C5—S1	120.5 (5)
Cu1—Cu2—I1	57.52 (3)	C4—C5—S1	116.2 (6)
I1^i^—Cu2—I1	114.41 (5)	N2—C6—C7	123.5 (6)
N2^i^—Cu2—I2	105.64 (15)	N2—C6—H6	118.3
Cu1—Cu2—I2	56.67 (3)	C7—C6—H6	118.3
I1^i^—Cu2—I2	107.23 (5)	C6—C7—C8	118.2 (6)
I1—Cu2—I2	104.38 (4)	C6—C7—H7	120.9
N2^i^—Cu2—Cu2^i^	133.76 (15)	C8—C7—H7	120.9
Cu1—Cu2—Cu2^i^	68.25 (5)	C9—C8—C7	119.9 (6)
I1^i^—Cu2—Cu2^i^	57.25 (4)	C9—C8—H8	120.0
I1—Cu2—Cu2^i^	57.17 (3)	C7—C8—H8	120.0
I2—Cu2—Cu2^i^	120.18 (6)	C8—C9—C10	118.0 (6)
C5—S1—S2	104.3 (2)	C8—C9—H9	121.0
C10—S2—S1	103.3 (2)	C10—C9—H9	121.0
C10—S2—Cu1	105.6 (2)	N2—C10—C9	123.4 (6)
S1—S2—Cu1	97.40 (10)	N2—C10—S2	114.0 (4)
C5—N1—C1	117.3 (6)	C9—C10—S2	122.6 (5)
C5—N1—Cu1	121.7 (5)		
			
Cu2^i^—I1—Cu1—N1	128.20 (18)	I1—Cu1—S2—C10	33.8 (2)
Cu2—I1—Cu1—N1	−160.76 (18)	I2—Cu1—S2—C10	169.2 (2)
Cu2^i^—I1—Cu1—S2	24.03 (6)	Cu2—Cu1—S2—C10	101.6 (2)
Cu2—I1—Cu1—S2	95.07 (7)	N1—Cu1—S2—S1	22.68 (17)
Cu2^i^—I1—Cu1—I2	−109.45 (5)	I1—Cu1—S2—S1	139.91 (8)
Cu2—I1—Cu1—I2	−38.41 (4)	I2—Cu1—S2—S1	−84.72 (9)
Cu2^i^—I1—Cu1—Cu2	−71.04 (4)	Cu2—Cu1—S2—S1	−152.27 (7)
Cu2—I2—Cu1—N1	163.59 (17)	S2—Cu1—N1—C5	−19.2 (5)
Cu2—I2—Cu1—S2	−102.17 (6)	I1—Cu1—N1—C5	−144.4 (4)
Cu2—I2—Cu1—I1	37.66 (4)	I2—Cu1—N1—C5	89.8 (5)
N1—Cu1—Cu2—N2^i^	−11.1 (6)	Cu2—Cu1—N1—C5	145.1 (4)
S2—Cu1—Cu2—N2^i^	152.3 (3)	S2—Cu1—N1—C1	169.7 (5)
I1—Cu1—Cu2—N2^i^	−90.1 (3)	I1—Cu1—N1—C1	44.5 (6)
I2—Cu1—Cu2—N2^i^	50.3 (3)	I2—Cu1—N1—C1	−81.3 (5)
N1—Cu1—Cu2—I1^i^	−167.0 (5)	Cu2—Cu1—N1—C1	−26.0 (9)
S2—Cu1—Cu2—I1^i^	−3.57 (6)	C5—N1—C1—C2	−0.4 (11)
I1—Cu1—Cu2—I1^i^	114.03 (5)	Cu1—N1—C1—C2	171.2 (6)
I2—Cu1—Cu2—I1^i^	−105.52 (5)	N1—C1—C2—C3	1.5 (12)
N1—Cu1—Cu2—I1	79.0 (5)	C1—C2—C3—C4	−0.9 (11)
S2—Cu1—Cu2—I1	−117.60 (7)	C2—C3—C4—C5	−0.7 (11)
I2—Cu1—Cu2—I1	140.45 (3)	C1—N1—C5—C4	−1.4 (10)
N1—Cu1—Cu2—I2	−61.4 (5)	Cu1—N1—C5—C4	−172.8 (5)
S2—Cu1—Cu2—I2	101.95 (7)	C1—N1—C5—S1	175.7 (5)
I1—Cu1—Cu2—I2	−140.45 (3)	Cu1—N1—C5—S1	4.3 (7)
N1—Cu1—Cu2—Cu2^i^	143.0 (5)	C3—C4—C5—N1	1.9 (11)
S2—Cu1—Cu2—Cu2^i^	−53.58 (6)	C3—C4—C5—S1	−175.3 (5)
I1—Cu1—Cu2—Cu2^i^	64.02 (4)	S2—S1—C5—N1	16.5 (6)
I2—Cu1—Cu2—Cu2^i^	−155.53 (5)	S2—S1—C5—C4	−166.2 (5)
Cu1—I1—Cu2—N2^i^	150.78 (17)	C10—N2—C6—C7	0.1 (9)
Cu2^i^—I1—Cu2—N2^i^	−125.65 (17)	Cu2^i^—N2—C6—C7	177.3 (5)
Cu2^i^—I1—Cu2—Cu1	83.57 (5)	N2—C6—C7—C8	1.2 (11)
Cu1—I1—Cu2—I1^i^	−83.57 (5)	C6—C7—C8—C9	−1.0 (10)
Cu2^i^—I1—Cu2—I1^i^	0.0	C7—C8—C9—C10	−0.3 (10)
Cu1—I1—Cu2—I2	33.31 (3)	C6—N2—C10—C9	−1.6 (9)
Cu2^i^—I1—Cu2—I2	116.88 (6)	Cu2^i^—N2—C10—C9	−178.5 (4)
Cu1—I1—Cu2—Cu2^i^	−83.57 (5)	C6—N2—C10—S2	−179.2 (4)
Cu1—I2—Cu2—N2^i^	−160.06 (14)	Cu2^i^—N2—C10—S2	4.0 (6)
Cu1—I2—Cu2—I1^i^	88.08 (5)	C8—C9—C10—N2	1.7 (10)
Cu1—I2—Cu2—I1	−33.68 (3)	C8—C9—C10—S2	179.1 (5)
Cu1—I2—Cu2—Cu2^i^	26.43 (5)	S1—S2—C10—N2	−157.5 (4)
C5—S1—S2—C10	83.7 (3)	Cu1—S2—C10—N2	−55.7 (5)
C5—S1—S2—Cu1	−24.3 (2)	S1—S2—C10—C9	25.0 (6)
N1—Cu1—S2—C10	−83.4 (3)	Cu1—S2—C10—C9	126.7 (5)

Symmetry codes: (i) −*x*+1, −*y*, −*z*+1.
